# HCAR score as a prognostic biomarker of survival in locally advanced nasopharyngeal carcinoma treated with concurrent chemoradiotherapy

**DOI:** 10.17305/bb.2025.13398

**Published:** 2025-12-05

**Authors:** Erkan Topkan, Efsun Somay, Duriye Ozturk, Ugur Selek

**Affiliations:** 1 Department of Radiation Oncology, Baskent University Medical Faculty, Adana, Türkiye; 2 Department of Oral and Maxillofacial Surgery, Faculty of Dentistry, Baskent University, Ankara, Türkiye; 3 Department of Radiation Oncology, Faculty of Medicine, Afyonkarahisar Health Sciences University, Afyonkarahisar, Türkiye; 4 Department of Radiation Oncology, Koc University School of Medicine, Istanbul, Türkiye

**Keywords:** Hemoglobins, C-reactive protein, albumins, biomarkers, prognosis, treatment outcome

## Abstract

Nasopharyngeal carcinoma (NPC) is an aggressive malignancy of the head and neck that is often diagnosed at a locally advanced stage (LANPC). In such cases, intensity-modulated radiotherapy (RT) combined with concurrent chemoradiotherapy (CCRT) is the standard treatment; however, the occurrence of distant metastasis and treatment failure remains prevalent. This study evaluates the prognostic significance of a novel composite score that combines hemoglobin levels and the C-reactive protein-to-albumin ratio (HCAR) in LANPC patients undergoing CCRT. We conducted a retrospective analysis of 233 LANPC patients treated with intensity-modulated RT and platinum-based CCRT from 2011 to 2020. Receiver operating characteristic curve analysis determined pretreatment hemoglobin (Hb) and C-reactive protein-to-albumin ratio (CAR) cut-offs of 11.0 g/dL and 3.0, respectively, which were utilized to create a three-tiered HCAR score: HCAR-0 (Hb ≥ 11.0 g/dL and CAR < 3.0), HCAR-1 (Hb ≥ 11.0 g/dL and CAR ≥ 3.0 or Hb < 11.0 g/dL and CAR < 3.0), and HCAR-2 (Hb < 11.0 g/dL and CAR ≥ 3.0). The primary endpoint of the study was overall survival (OS), while progression-free survival (PFS) was the secondary endpoint. With a median follow-up of 85.7 months, the median PFS and OS were 66.0 months and 108.0 months, respectively, with 5-year PFS and OS rates of 52.8% and 75.9%. The HCAR score significantly stratified patient outcomes: median PFS was not reached for HCAR-0, 66.0 months for HCAR-1, and 25.0 months for HCAR-2. Median OS also varied significantly, being not reached for HCAR-0, 108.0 months for HCAR-1, and 55.0 months for HCAR-2 (all *p* < 0.001). Corresponding 10-year PFS rates were 50.2%, 34.4%, and 5.0%, while 10-year OS rates were 68.3%, 41.6%, and 11.1%. Multivariate analysis revealed that the HCAR score remained an independent predictor of both PFS and OS, alongside T and N stage. The HCAR score shows promising prognostic utility for predicting OS and PFS in LANPC; however, performance estimates may be overly optimistic due to the lack of internal validation.

## Introduction

Nasopharyngeal carcinoma (NPC) is an aggressive malignancy of the head and neck, with a notably high prevalence in Southeast Asia and southern China [1]. The global distribution of NPC is markedly uneven, displaying incidence rates of 20–30 cases per 100,000 individuals in East and Southeast Asia, yet it ranks only 24th in global cancer incidence and 22nd in mortality [2, 3]. Due to the absence of early symptoms, approximately 70% of patients are diagnosed at the locally advanced stage (LANPC) [4]. Surgical intervention is limited in NPC management due to the anatomical constraints of the nasopharyngeal region and is primarily reserved for locoregional recurrences or persistent neck disease [5]. For early-stage NPC, radiotherapy (RT) alone remains the preferred treatment approach. Conversely, for LANPC, the tumor’s high intrinsic radiosensitivity and chemosensitivity have established platinum-based concurrent chemoradiotherapy (CCRT) with intensity-modulated RT (IMRT) as the standard of care [6–9]. IMRT-based CCRT has significantly improved toxicity profiles, locoregional control, and survival rates. Nonetheless, the overall 5-year survival rate for LANPC remains approximately 80% [10], with distant metastasis (DM) and, to a lesser extent, local and/or regional recurrences continuing to be the primary causes of treatment failure and mortality [11].

The tumor-node-metastasis (TNM) staging system is currently the gold standard for prognostic stratification and treatment decision-making for patients with NPC [12]. However, it is frequently observed that patients with LANPC exhibit significantly varied outcomes despite receiving equivalent treatments for comparable stages of the disease [12–14]. These substantial variations in outcomes among patients diagnosed with identical stages of LANPC are primarily due to the limitations inherent in the TNM staging system. This system fails to account for biological differences among tumors and their respective hosts, as it relies exclusively on the local and regional progression of the primary tumor and the associated lymph nodes [15, 16]. Therefore, these shortcomings in the TNM staging framework underscore the urgent need to identify more relevant and innovative prognostic factors and potentially incorporate them into future staging systems.

Over half of all solid tumors contain hypoxic regions—termed tumor hypoxia—which serve as a well-established surrogate marker of resistance to both RT and chemotherapy across various cancer types, including NPC [17, 18]. In irradiated cancer cells, molecular oxygen interacts with radiation-induced DNA radicals, effectively “fixing” the damage and preventing subsequent DNA repair, provided that adequate oxygen levels are present. Conversely, under hypoxic conditions, this oxygen fixation process is impaired, allowing for effective repair of DNA lesions and conferring radioresistance to the tumor cell population. In addition to impairing radiosensitivity, tumor hypoxia promotes the stabilization of hypoxia-inducible factors (HIFs), which drive neoangiogenesis, genomic instability, and the accumulation of genetic mutations. These changes foster the emergence of aggressive tumor phenotypes that exhibit marked resistance to apoptosis induced by reactive oxygen species [17–19]. Clinical studies have consistently demonstrated that anemia and hemoglobin (Hb) levels below 11.0 g/dL are significantly associated with reduced median and long-term survival in patients with LANPC undergoing definitive CCRT [20–23].

Three emerging factors closely linked to the prognosis of LANPC are the patient’s nutritional, immune, and systemic inflammatory status. Two readily available and cost-effective biochemical parameters that simultaneously reflect these aspects are C-reactive protein (CRP) and serum albumin. The CRP-to-albumin ratio (CAR), a novel inflammation-based prognostic index, has demonstrated substantial prognostic value in patients with NPC [24, 25]. Elevated CAR values have consistently been associated with unfavorable outcomes across various treatment modalities and disease stages, including LANPC [24, 25]. While both Hb and CAR have been studied independently as prognostic markers in patients with LANPC, the potential of combining these two parameters into a single composite score has yet to be explored. Building on previous evidence supporting the individual prognostic significance of Hb and CAR, we hypothesized that integrating them into a unified score—referred to as the HCAR (Hb–CAR) score—could enhance prognostic discrimination compared to either parameter alone. To evaluate this hypothesis, we conducted a retrospective analysis to determine the predictive value of the HCAR score in LANPC patients who underwent definitive CCRT followed by adjuvant chemotherapy.

## Materials and methods

### Study population, ethics, and consent

The research protocol received approval from the Institutional Review Board (IRB) of Baskent University Medical Faculty (Project No: D-KA-2058) prior to the initiation of patient data collection. This study adhered to the ethical standards established by the Declaration of Helsinki and its subsequent revisions. All participants who met the eligibility criteria were thoroughly informed about the study’s objectives and voluntarily provided signed informed consent. This consent encompassed permission for the collection and analysis of patient and disease characteristics, blood samples, and pathology specimens, as well as the anonymous dissemination of research findings in academic venues.

### Study design and participants

This study followed the reporting recommendations outlined by the REMARK guidelines [26]. We conducted a single-center, retrospective cohort study at the Department of Radiation Oncology at Baskent University Medical Faculty. Our investigation involved a comprehensive review of medical records for patients diagnosed with LANPC who underwent CCRT at our institution from June 2011 to December 2020.

Eligible patients were required to meet the following criteria: age between 18 and 80 years; Eastern Cooperative Oncology Group (ECOG) performance status of 0–2; and a body mass index (BMI) ≥ 18.5 kg/m^2^. Pretreatment evaluations included a complete clinical ear–nose–throat (ENT) examination, head and neck magnetic resonance imaging (MRI), and fluorodeoxyglucose-positron emission computed tomography (FDG-PET/CT). Disease staging was based on the 8th edition of the American Joint Committee on Cancer (AJCC) staging system, including clinical or radiological stages T3-4N0-3M0 or T1-4N1-3M0. Histopathological confirmation of non-keratinizing or undifferentiated squamous cell carcinoma was mandatory. World Health Organization (WHO) type I keratinizing NPC was excluded due to its rarity in our population and its well-documented biological and prognostic distinctions from WHO type II/III tumors.

Patients were excluded if they had a history of other malignancies, received prior chemotherapy or RT, had active infectious diseases, or used immunosuppressive drugs within 30 days before starting CCRT. All included patients were required to have received at least one cycle of platinum-based concurrent chemotherapy. Additionally, the availability of complete records for RT and chemotherapy, baseline complete blood count results, and follow-up ENT examinations, MRI, and PET-CT scans was necessary for inclusion.

### Chemoradiotherapy protocol

All research participants underwent definitive CCRT with established dosages of RT and chemotherapy [22]. Patients were treated with simultaneous integrated boost–IMRT (SIB-IMRT) following a uniform institutional protocol. Target volumes were defined according to contemporary international guidelines for NPC. The gross tumor volume (GTV) included the primary nasopharyngeal tumor and any involved lymph nodes identified on MRI and/or PET-CT. The high-risk clinical target volume (CTV1) encompassed the GTV with a 5–10 mm margin adjusted to anatomical boundaries, incorporating regions at the highest risk for microscopic extension. The low-risk clinical target volume (CTV2) included bilateral cervical nodal regions at risk but without radiological involvement. Planning target volumes (PTVs) were generated by expanding each CTV by 3–5 mm to account for setup uncertainties. A three-level SIB-IMRT prescription was utilized for all patients: 70 Gy to PTV-GTV, 60 Gy to PTV-CTV1, and 54 Gy to PTV-CTV2, delivered in 33 fractions over 6.5 weeks. Treatment planning was exclusively conducted using the Varian Eclipse treatment planning system with 6-MV photon beams. Dose constraints adhered to departmental standards, including maximum allowable doses of <45 Gy to the spinal cord, <54 Gy to the brainstem and optic chiasm, and a mean parotid dose of ≤26 Gy whenever feasible. Constraints for the temporal lobes, cochleae, mandible, and other organs at risk were established according to international guidelines for NPC IMRT. Daily image guidance was performed using kilovoltage (kV) orthogonal imaging and/or cone-beam CT to ensure accurate patient positioning and reproducibility throughout the treatment course. All patients received concurrent cisplatin at a dosage of 75–80 mg/m^2^ every three weeks; adjuvant chemotherapy was administered for two cycles when tolerated. Appropriate supportive care interventions, including oral or intravenous hydration, antiemetic agents, and oral or enteral nutritional supplements, were provided as clinically indicated.

### Measurement of Hb and CAR: Construction of the HCAR score

Pretreatment Hb, CRP, and albumin levels were measured on the first day of CCRT using a standardized automated chemistry analyzer (Beckman Coulter AU-series). CRP values were reported in mg/L and albumin in g/L, with internal quality control (QC) procedures performed daily in accordance with manufacturer standards (typical institutional QC ranges: CRP 0–5 mg/L; albumin 35–50 g/L). The CAR was calculated as: CRP (mg/L) ÷ albumin (g/L).

### Response assessment

This study utilized a retrospective design; however, the evaluation of treatment response was conducted prospectively in accordance with institutional protocols. During the first two years of follow-up, patients underwent clinical and radiological assessments every three months. This frequency transitioned to every six months during years three through five and subsequently shifted to annual evaluations, or more frequently if clinically necessary. Endoscopic examinations were routinely performed at each follow-up to detect local or regional tumor recurrences as well as any second primary tumors in the head and neck region. To assess treatment response and identify potential distant metastases, PET-CT scans were utilized based on the PET Response Criteria for Solid Tumors (PERCIST). Once a complete metabolic response was established, subsequent imaging surveillance primarily included MRI and/or CT scans of the head and neck rather than PET-CT scans. Additional imaging techniques were employed to investigate suspicious lesions or to reassess cases where tumor recurrence was suspected.

### Clinical endpoints and statistics

The primary objective of this study was to evaluate the prognostic significance of the HCAR score for overall survival (OS) in patients with LANPC treated with CCRT. OS was calculated from the initiation of CCRT to death from any cause, with patients who remained alive at the last contact censored at their most recent follow-up visit. The secondary objective was to assess the relationship between the HCAR score and progression-free survival (PFS), defined as the period from the onset of CCRT to the first documented relapse or death. Patients without events were right-censored at the date of their last clinical contact.

Continuous variables were summarized using medians and ranges, while categorical variables were described as frequency distributions. Baseline characteristics were compared using appropriate statistical tests: chi-square or Fisher’s exact tests for categorical variables (based on expected cell frequencies) and Student’s *t*-tests or non-parametric aligned rank tests for continuous variables, depending on distributional assumptions. Effect sizes (mean differences or risk ratios) with corresponding 95% confidence intervals (CIs) were calculated to complement *P* values and enhance clinical interpretability.

Receiver operating characteristic (ROC) curve analysis was conducted to determine the optimal pre-CCRT Hb and CAR cut-off values that maximized the separation of OS and PFS outcomes. All ROC analyses were performed using conventional status-based (binary outcome) ROC, with OS or PFS status at the last follow-up serving as the classifier, rather than time-dependent ROC. Kaplan–Meier curves and log-rank tests were utilized to examine associations between clinical or biomarker variables and survival outcomes. Variables demonstrating significance in univariate analysis were subsequently entered into multivariable Cox proportional hazards (PH) models to evaluate their independent prognostic contributions. For all comparisons, a two-tailed *P* value of <0.05 was considered statistically significant. Bonferroni-adjusted *P* values were employed for comparisons involving three or more groups, including analyses across HCAR score categories, to mitigate the risk of false-positive findings.

Diagnostics for the Cox PH models were conducted to assess model adequacy. The PH assumption was evaluated using Schoenfeld residual-based tests and visual inspections of log–log survival plots. The functional form of continuous variables (Hb, CAR, and age) was examined using residual-based diagnostics to detect non-linearity. Influential observations were assessed using dfbeta statistics to identify cases exerting disproportionate influence on model coefficients. Model discrimination was quantified using Harrell’s C-index with 95% CIs, and calibration was assessed using the calibration slope and intercept. Additional details are provided in Supplementary Material 1.

A total of 105 PFS events and 83 OS events contributed to the multivariable Cox models. To further ensure the adequacy of these models, we documented the number of events contributing to each Cox model and evaluated the corresponding events-per-variable (EPV). All models met commonly accepted EPV thresholds for exploratory prognostic Cox regression, supporting the stability of coefficient estimation and minimizing the risk of overfitting. As only essential covariates were included and EPV criteria were satisfied, penalized Cox regression was not necessary for this study.

The assessment of incremental prognostic value using nested Cox models (e.g., Hb-only, CAR-only, HCAR-only, or HCAR + TNM) was not performed due to the insufficient number of available events to support the additional model complexity. Although the primary multivariable model met acceptable EPV criteria, constructing multiple nested alternatives would have significantly reduced EPV, resulting in unstable coefficient estimates and an increased risk of overfitting. Under these constraints, likelihood-ratio testing, ΔC-index estimation, and AIC-based comparisons would not yield reliable or interpretable incremental-value estimates. Therefore, nested-model evaluations are deferred to future studies with larger event counts.

All statistical analyses were conducted using IBM SPSS Statistics version 26 (IBM Corp., Armonk, NY, USA).

### Ethics approval and consent to participate

Prior to collecting any data from patients, the study design received approval from the IRB of Baskent University School of Medicine and adhered to the Declaration of Helsinki.

## Results

A total of 233 patients with LANPC who underwent definitive CCRT were eligible for inclusion (Table 1). The median age at presentation was 57 years (range, 19–79 years), with 33.5% of patients aged 65 years or older. Males comprised the majority of the cohort (80.0%). All patients had an ECOG performance status of 0 (47.6%) or 1 (52.4%). Histologically, most patients (87.1%) were diagnosed with WHO type III undifferentiated carcinoma, while the remainder had WHO type II. The majority presented with advanced primary and nodal disease, with 81.1% staged as T3–4 and 78.1% staged as N2–3. Treatment compliance was relatively high (Table 1). A total of 72.5% of patients completed all three prescribed cycles of concurrent cisplatin, while 27.5% received one or two cycles of treatment. Following CCRT, 73.0% of patients proceeded to adjuvant chemotherapy, with the majority receiving two cycles (58.4%). The median interval between pathological diagnosis and the initiation of CCRT was 18 days (range: 9–28).

**Table 1:** Baseline demographic, clinical, and treatment-related characteristics of the entire study cohort and according to HCAR groups

**Characteristics**	**Whole cohort** **(*n* ═ 233)**	**HCAR-0** **(*n* ═ 88)**	**HCAR-1** **(*n* ═ 91)**	**HCAR-2** **(*n* ═ 54)**	***P* value**
Median age, years (range)	57 (19–79)	58 (27–79)	57 (28–79)	56 (19–77)	0.86
*Age group (N; %)*					
≥65 years <65 years	78 (33.5) 155 (66.5)	31 (35.2) 57 (64.8)	33 (36.3) 58 (63.7)	14 (25.9) 40 (74.1)	0.19
*Gender (N; %)*					
Male Female	184 (80.0) 49 (20.0)	71 (80.7) 17 (19.3)	71 (78.0) 20 (22.0)	42 (77.8) 12 (22.2)	0.86
*ECOG performance (N; %)*					
0 1	111 (47.6) 122 (52.4)	43 (48.9) 45 (51.1)	46 (50.5) 45 (49.5)	22 (40.7) 32 (59.3)	0.28
*WHO histology (N; %)*					
2 3	30 (12.9) 203 (87.1)	12 (13.6) 76 (86.4)	11 (12.1) 80 (87.9)	7 (13.0) 47 (87.0)	0.84
*T-stage (N; %)*					
1–2 3–4	44 (18.9) 189 (81.1)	17 (19.3) 71 (80.7)	18 (19.8) 73 (80.2)	9 (16.7) 45 (83.3)	0.63
*N-stage (N; %)*					
0–1 2–3	51 (21.9) 182 (78.1)	18 (20.5) 70 (79.5)	20 (22.0) 71 (78.0)	13 (24.1) 41 (75.9)	0.57
*Concurrent chemotherapy cycles (N; %)*					
1–2 3	64 (27.5) 169 (72.5)	24 (27.3) 64 (72.7)	25 (27.5) 66 (72.5)	15 (27.8) 39 (72.2)	0.87
*Adjuvant chemotherapy cycles (N; %)*					
0 1–2	63 (27.0) 170 (73.0)	25 (28.4) 63 (71.6)	26 (28.6) 65 (71.4)	12 (22.2) 42 (77.8)	0.42

The Bonferroni corrected *P* value is significant at <0.0167. Corresponding effect size estimates, along with their 95% confidence intervals, are detailed in Table S3. Abbreviations: HCAR: Combination of Hb and the CAR; Hb: Hemoglobin; CAR: C-reactive protein-to-albumin ratio; ECOG: Eastern Cooperative Oncology Group; WHO: World Health Organization; T-stage: Tumor stage; N-stage: Nodal stage.

At a median follow-up of 85.7 months (95% CI, 67.9–103.5), 150 patients (64.4%) were alive, and 128 (54.9%) remained free of disease progression. Local control was achieved in 212 patients (91.4%). The median PFS was 66.0 months (95% CI, 53.6–78.4), with 5- and 10-year PFS rates of 52.8% and 31.6%, respectively. The median OS was 108.0 months (95% CI, 92.3–123.7), with corresponding 5- and 10-year OS rates of 75.9% and 43.1% (Table 2).

**Table 2:** Survival outcomes for the overall study population and groups categorized by HCAR score

**Outcome**	**Whole cohort** **(*n* ═ 233)**	**HCAR-0** **(*n* ═ 88)**	**HCAR-1** **(*n* ═ 91)**	**HCAR-2** **(*n* ═ 54)**	***P* value**
*Progression-free survival*					
Median; mo. (95% CI) 5-year (%) 10-year (%)	66.0 (53.6–78.4) 52.8 31.6	NR 66.0 50.2	66.0 (50.6–81.4) 52.2 34.4	25.0 (17.8–32.2) 33.3 5.0	<0.001
*Overall survival*					
Median; mo. (95% CI) 5-year (%) 10-year (%)	108.0 (92.3–123.7) 75.9 43.1	NR 83.7 68.3	108.0 (89.3–126.7) 78.5 41.6	55.0 (43.8–66.2) 48.3 11.1	<0.001

Significant Bonferroni corrected *P* value: <0.0167. Abbreviations: HCAR: Combination of hemoglobin (Hb) and the C-reactive protein-to-albumin ratio (CAR); CI: Confidence interval; mo: Months; NR: Not reached.

**Figure 1.**
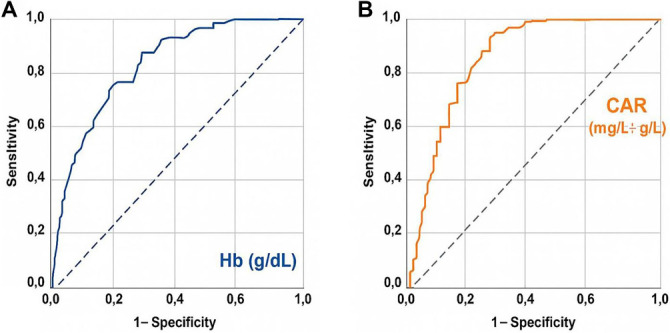
**ROC curve analysis assessing the relationship between OS and pretreatment biomarkers.** (A) The association between pretreatment Hb levels and OS status, and (B) the relationship between the pretreatment CAR and OS status. PFS-specific ROC performance is detailed in Table S1. Abbreviations: ROC: Receiver Operating Characteristic; OS: Overall survival; Hb: Hemoglobin; CAR: C-reactive protein-to-albumin ratio; PFS: Progression-free survival.

**Figure 2.**
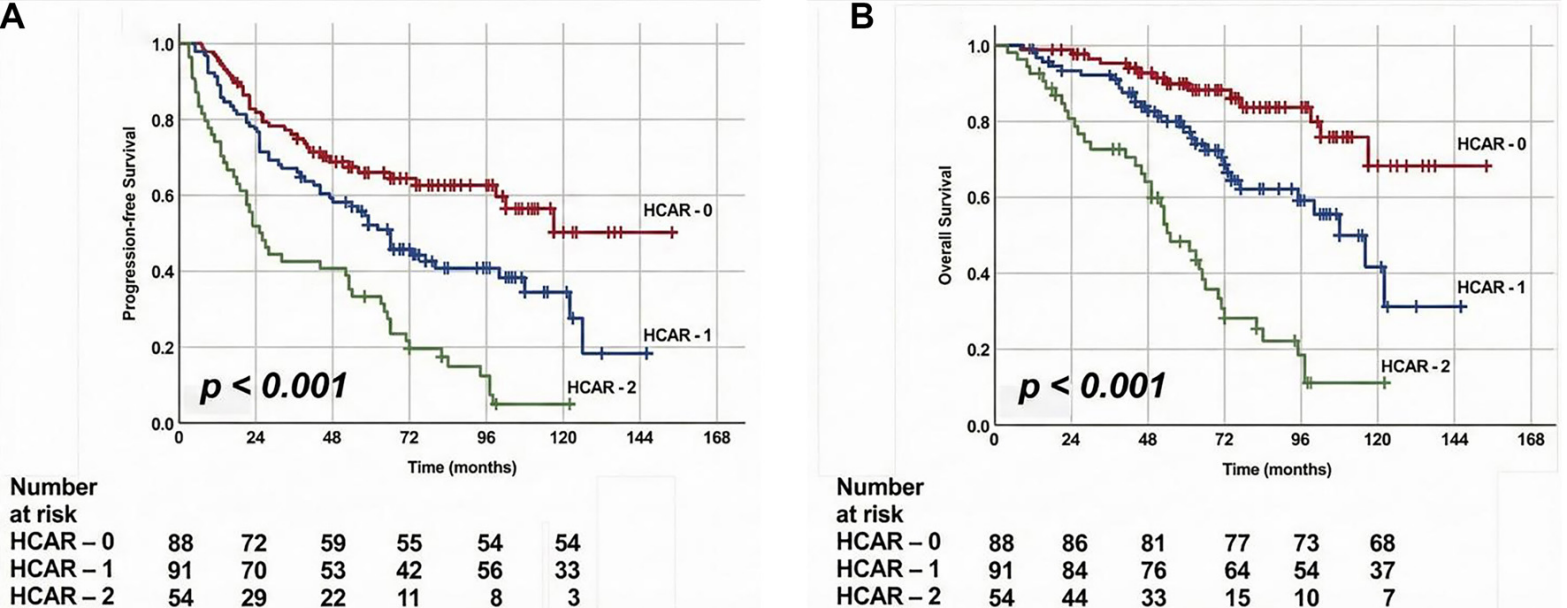
**Kaplan–Meier curves for (A) progression-free survival and (B) overall survival according to the three-tiered HCAR score.** The HCAR score was derived from pretreatment Hb and CAR using cut-offs of 11.0 g/dL and 3.0, respectively, and categorized as HCAR-0 (Hb ≥ 11.0 and CAR < 3.0), HCAR-1 (Hb ≥ 11.0 and CAR ≥ 3.0 or Hb < 11.0 and CAR < 3.0), and HCAR-2 (Hb < 11.0 and CAR ≥ 3.0). Both progression-free and overall survival differed significantly across HCAR groups (log-rank *P* < 0.001). Numbers at risk at selected time points are shown below each plot. Abbreviations: HCAR: Combination of Hb and the CAR; Hb: Hemoglobin; CAR: C-reactive protein-to-albumin ratio; PFS: Progression-free survival; OS: Overall survival.

ROC curve analyses were performed for each biomarker–endpoint pair, with results summarized in Table S1. For Hb, the optimal cut-off for OS was found to be 11.1 g/dL (AUC: 0.816, sensitivity: 77.9%, specificity: 76.8%, Youden J: 0.547), while the optimal cut-off for PFS was 10.9 g/dL (AUC: 0.789, sensitivity: 75.3%, specificity: 72.4%, Youden J: 0.477). For CAR, the OS-specific cut-off was 2.95 (AUC: 0.872, sensitivity: 82.1%, specificity: 76.1%, Youden J: 0.582), and the PFS-specific cut-off was 3.10 (AUC: 0.804, sensitivity: 78.3%, specificity: 75.2%, Youden J: 0.535) (Figure 1). Due to the proximity of these OS- and PFS-specific Hb and CAR values, we selected rounded thresholds of 11.0 g/dL for Hb and 3.0 for CAR for subsequent analyses. This resulted in the creation of four possible HCAR score groups: HCAR-0: Hb ≥ 11.0 and CAR < 3.0; HCAR-1: Hb ≥ 11.0 and CAR ≥ 3.0; HCAR-2: Hb < 11.0 and CAR < 3.0; and HCAR-3: Hb < 11.0 and CAR ≥ 3.0. However, comparisons among the four groups revealed no statistically significant differences in either PFS or OS between the original HCAR-1 and HCAR-2 groups (Table S2), leading to their merger and the establishment of final three-tiered HCAR score groups: HCAR-0 (Hb ≥ 11.0 and CAR < 3.0), HCAR-1 (Hb ≥ 11.0 and CAR ≥ 3.0 or Hb < 11.0 and CAR < 3.0), and HCAR-2 (Hb < 11.0 and CAR ≥ 3.0). Comparative survival analyses indicated that the median PFS (not reached vs 66.0 vs 25.0 months; *P* < 0.001) and median OS (not reached vs 108.0 vs 55.0; *P* < 0.001) durations were significantly longer in the HCAR-0 group, with the HCAR-2 group exhibiting the shortest durations and the HCAR-1 group positioned in between (Table 2 and Figure 2). Corresponding 5-year and 10-year PFS and OS rates were also more favorable in the HCAR-0 group compared to the HCAR-1 and HCAR-2 groups. Similarly, the HCAR-1 group demonstrated superior 5-year and 10-year PFS and OS rates compared to the HCAR-2 group (Table 2). Importantly, these findings occurred without any statistically significant imbalances in baseline demographic, clinical, or treatment-related variables across the three HCAR groups (all Bonferroni corrected *P* > 0.0167; Table 1). Additionally, effect sizes with 95% CIs were uniformly small, indicating no clinically meaningful baseline imbalances across HCAR groups, further supporting cohort comparability (Table S3). Notably, there were no significant differences in treatment adherence among the HCAR categories concerning concurrent (*P* ═ 0.87) and adjuvant chemotherapy cycles (*P* ═ 0.42), suggesting that the observed outcome differences are not attributable to variations in treatment compliance.

In the univariate analysis, T stage (PFS: *P* ═ 0.008; OS: *P* ═ 0.003), N stage (PFS: *P* < 0.001; OS: *P* < 0.001), and HCAR score (PFS: *P* < 0.001; OS: *P* < 0.001) were identified as significant prognostic indicators. Conversely, factors such as age, gender, ECOG performance status, histology, and the number of cycles of concurrent or adjuvant chemotherapy showed no correlation with outcomes (Table 3). Furthermore, multivariate Cox regression analysis confirmed that HCAR score, T stage, and N stage serve as independent prognostic factors for both PFS and OS (Table 3).

**Table 3:** Univariate and multivariate Cox regression analyses of prognostic factors for progression-free survival and overall survival

	**PFS**			**OS**		
**Factor**	**Univariate** ***P* value**	**Multivariate** ***P* value**	**HR** **(95% CI)**	**Univariate** ***P* value**	**Multivariate** ***P* value**	**HR** **(95% CI)**
Age group (<65 vs ≥65 y)	0.63	–	–	0.57	–	–
Gender (Male vs Female)	0.69	–	–	0.61	–	–
ECOG (0 vs 1)	0.88	–	–	0.82	–	–
WHO histology (2 vs 3)	0.76	–	–	0.63	–	–
T-stage (1–2 vs 3–4)	0.008	0.011	0.65 (0.42–0.83)	0.003	0.009	0.52 (0.33–0.81)
N-stage (0–1 vs 2–3)	<0.001	0.001	0.47 (0.37–0.78)	<0.001	0.001	0.44 (0.22–0.69)
Concurrent chemotherapy cycles (1–2 vs 3)	0.27	–	–	0.22	–	–
Adjuvant chemotherapy cycles (0 vs 1–2)	0.43	–	–	0.49	–	–
*HCAR group (reference = HCAR-0)^a^*						
1 vs 0 2 vs 0 2 vs 1	0.012 <0.001 <0.001	0.014 <0.001 <0.001	1.76 (1.34–2.93) 7.69 (5.17–11.84) 3.76 (2.28–4.98)	0.007 <0.001 <0.001	0.011 <0.001 <0.001	3.06 (1.89–5.12) 9.17 (6.12–15.62) 5.84 (4.18–7.26)

Events included in the Cox models: 105 PFS events; 83 OS events. ^a^Significant Bonferroni corrected *P* value: <0.0167. Abbreviations: PFS: Progression-free survival; OS: Overall survival; HR: Hazard ratio; CI: Confidence interval; ECOG: Eastern Cooperative Oncology Group; WHO: World Health Organization; T-stage: Tumor stage; N-stage: Nodal stage; HCAR score = combination of hemoglobin (Hb) and the C-reactive-protein-to-albumin ratio (CAR).

Given the markedly different prognostic strengths of the anatomic components in our cohort—N stage exhibiting a highly significant association with PFS (*P* < 0.001), while T stage demonstrated a much weaker effect (*P* ═ 0.011)—the combined AJCC/UICC stage group was excluded from the multivariate model. Since all patients presented with M0 disease, the stage group serves as a deterministic composite of T and N; including this variable would introduce structural collinearity and obscure the independent contribution of each component. This well-known masking phenomenon, where the more decisive factor (*N*) accounts for most of the shared variance, can lead to unstable or biased hazard estimates. For this reason, stage distributions are presented descriptively in Table 1, while only variables demonstrating independent prognostic value without collinearity concerns were retained in the final multivariable analysis. Importantly, in univariate analyses (Kaplan–Meier and univariate Cox), AJCC/UICC stage group exhibited patterns fully consistent with the separate T- and N-stage effects, with N stage functioning as the principal prognostic determinant; therefore, no prognostic information was lost by excluding the stage group from the multivariable model. Model performance assessment demonstrated acceptable discrimination (PFS C-index: 0.71, OS C-index: 0.73) and good calibration for both multivariable models; full metrics are provided in Supplementary Material 1.

## Discussion

In this retrospective cohort analysis, our results demonstrate that the novel three-tiered HCAR score, derived from baseline Hb levels and calculated CAR values, serves as an independent prognostic biomarker for both PFS and OS in patients with LANPC treated with definitive CCRT. This study is the first to integrate a host-related hypoxia surrogate (Hb) with an immune-inflammation-nutrition index (CAR) into a composite score and systematically evaluate its prognostic value in this patient population.

Our findings reaffirm the established prognostic significance of T and N stages, which together form the basis of the TNM staging system in non-metastatic NPC [27]. However, notable heterogeneity in survival outcomes is frequently observed among LANPC patients at the same disease stage, despite receiving similar CCRT regimens [22, 28]. In our study, the 95% CIs for PFS ranged from 53.6 to 78.4 months, and for OS, from 92.3 to 123.7 months, highlighting a disparity of 24.8 and 31.4 months, respectively, between the lower and upper limits of the 95% CI for these survival endpoints. These variations indicate that anatomical staging alone is insufficient to capture the complex biological and host-related factors influencing treatment responses and outcomes [15, 16, 29]. Therefore, there is an urgent need to incorporate additional prognostic markers that accurately reflect tumor biology and the specific systemic conditions of patients. While both Hb and CAR have been independently validated as prognostic factors in LANPC, no prior study has combined these parameters into a cohesive scoring system [20–25]. Consequently, we developed the HCAR score, an innovative composite biomarker that integrates Hb, a surrogate for tumor oxygenation and radiosensitivity, with CAR, a recognized indicator of systemic inflammation, immune competence, and nutritional status. This aims to enhance the accuracy of outcome predictions in these patients.

The primary contribution of this study is the introduction of the HCAR score, the first prognostic model to integrate Hb and CAR into a single, clinically accessible composite index for LANPC patients. The HCAR scoring system effectively stratified patients into three distinct prognostic groups with markedly different PFS and OS outcomes: HCAR-0 patients exhibited excellent long-term survival (10-year OS 68.3%), HCAR-2 patients displayed poor outcomes (10-year OS 11.1%), and HCAR-1 patients demonstrated intermediate survival (10-year OS 41.6%). These findings underscore the additive prognostic value of capturing systemic oxygenation, inflammatory, immune, and nutritional status in LANPC patients. Importantly, the prognostic significance of the HCAR score was independent of T and N stages, and the observed survival differences were not attributable to baseline clinicopathologic imbalances or variations in the intensity of concurrent or adjuvant chemotherapy. Collectively, these results suggest that the HCAR score may serve as a practical complement to conventional TNM staging, facilitating more refined risk stratification and informing personalized therapeutic strategies for patients with LANPC.

Interpreting these novel findings in relation to the existing literature on LANPC is challenging due to the absence of prior research examining the prognostic implications of the HCAR score in this population. Nevertheless, these findings are consistent with earlier reports on the prognostic relevance of Hb and CAR, which together constitute the HCAR score, in similar LANPC cohorts [20–25]. For instance, Topkan and colleagues demonstrated that pre-CCRT Hb levels below 11.0 g/dL were independently associated with significantly inferior median OS (*P* < 0.001), PFS (*P* < 0.001), and locoregional PFS (*P* ═ 0.004) [22]. Notably, this association was notably stronger than that observed after categorizing patients according to the anemia cut-offs established by the WHO [30]. These findings were later corroborated by a study conducted by Cobanoglu et al. [31]. Likewise, Guo et al. [21] showed that Hb levels, measured both before and during CCRT and categorized as anemic or non-anemic, are significant prognostic markers for survival outcomes in NPC patients treated with IMRT. Considering the implications of the CAR, prior studies on NPC patients treated with definitive RT have consistently demonstrated a strong association between elevated pretreatment CAR values and unfavorable clinical outcomes. For example, Tao et al. [32] reported that NPC patients undergoing IMRT with high CAR values had significantly lower 5-year OS compared to those with low CAR values (78.1% vs 91.9%; *P* < 0.001). Similarly, a meta-analysis by Yang et al. [25] confirmed that elevated pretreatment CAR is an adverse prognostic indicator, showing significant correlations with both inferior OS (HR = 1.58, 95% CI = 1.36–1.83, *P* < 0.001) and reduced DM-free survival (HR = 1.25, 95% CI = 1.09–1.44, *P* ═ 0.002). Taken together, the available research results underscore the prognostic utility of Hb and CAR, providing a compelling rationale for their integration into a single composite index, namely the HCAR score, to enhance risk stratification and guide treatment personalization in NPC. However, confirmatory evidence from rigorously designed studies is needed before the HCAR score can be adopted as an adjunct to the TNM staging system for patients with LANPC.

The precise biological mechanisms linking an elevated HCAR score to poorer PFS and OS remain to be fully elucidated. However, prior research has highlighted the critical roles of its components—Hb, albumin, and CRP—in tissue oxygenation, nutritional status, systemic inflammation, and immune regulation [17–25]. These factors have been associated with tumor growth facilitation, metastatic spread, treatment resistance, and ultimately poorer prognosis, which may explain the unfavorable outcomes observed in patients with higher HCAR scores [25, 33–35]. Nevertheless, the clinical implications of our findings may be substantial, as Hb and CAR are inexpensive, routinely available laboratory parameters, allowing the HCAR score to be easily incorporated into clinical practice without the need for additional testing. Importantly, the incremental prognostic value of the HCAR score beyond Hb or CAR individually was not quantified in this exploratory dataset; whether the HCAR score offers additional discrimination or reclassification benefits over single-marker models remains to be determined in larger studies with sufficient statistical power. By stratifying patients into three prognostic categories, the score can assist in tailoring treatment—escalating therapy, increasing surveillance, or adding novel agents for high-risk patients (HCAR-2), while identifying low-risk patients (HCAR-0) who may achieve excellent outcomes with standard CCRT and avoid unnecessary intensification. Its prognostic independence from T and N stages further supports its role as an additional stratification factor in future clinical trials, enabling risk-adapted designs and more precise evaluations of novel therapies in LANPC. If prospectively validated in large, multi-institutional cohorts, the HCAR score could provide a practical step toward more personalized and biologically informed patient management.

This study has several limitations. First, its retrospective, single-institution design introduces potential selection bias, although baseline characteristics were generally balanced across the HCAR groups. Second, while the cohort size and follow-up duration were adequate, external validation in independent, multi-institutional datasets is required to confirm generalizability. Third, Hb and CAR are dynamic biomarkers that may fluctuate during treatment; our analysis relied solely on baseline measurements, and future prospective studies should evaluate the prognostic relevance of on-treatment or post-treatment changes in the HCAR score. Fourth, despite the biological rationale for integrating Hb and CAR, mechanistic studies are needed to clarify how different HCAR strata influence NPC progression and resistance to CCRT. Fifth, Hb and CAR were dichotomized using ROC-derived cut-offs rather than modeled as continuous predictors through flexible methods such as restricted cubic splines. While this approach enabled the construction of a simple, clinically practical score, it may not fully capture the continuous nature of these biomarkers. Additionally, EBV DNA levels, LDH, smoking status, and comorbidity indices were not uniformly available and could not be incorporated into sensitivity analyses, representing another limitation. Larger multicenter cohorts should explore spline-based modeling, coefficient-derived scoring, and formal internal validation (e.g., bootstrap optimism-correction, calibration assessment, and cross-validated C-indices) to determine whether refined HCAR frameworks offer superior prognostic performance. Given our dataset’s limited number of events and its original design not intended for complete model construction, rigorous internal validation and nested-model comparisons could not be performed without risking model instability. Consequently, some degree of optimism is expected regarding ROC-derived cut-offs and performance estimates, and the present findings should be interpreted as preliminary until confirmed in larger, independently validated cohorts.

## Conclusion

In conclusion, this study introduces the HCAR score as a novel composite biomarker capable of effectively stratifying LANPC patients into three distinct prognostic groups prior to the initiation of definitive CCRT. By simultaneously reflecting host hypoxia status, systemic immune response, inflammation levels, and nutritional reserve, the HCAR score has the potential to enhance risk stratification when used alongside the traditional TNM staging system, pending validation through future research.

6

**Consent for publication:** All patients were required to sign an informed consent form prior to the evaluation. This consent could be provided by the patients themselves or by their legally authorized representatives. The consent covered the acquisition and analysis of the patients’ sociodemographic, dental, and medical records, as well as the collection of blood samples and the publication of the findings.

## Supplemental data

Supplemental data are available at the following link: https://www.bjbms.org/ojs/index.php/bjbms/article/view/13398/4069.

## Data Availability

Data cannot be shared publicly because it is owned and saved by the Baskent University Medical Faculty. However, for researchers who meet the criteria for access to confidential data, data are available from the Baskent University Institutional Data Access/Ethics Committee (contact via Baskent University Ethics Committee): contact address, adanabaskent@baskent.edu.tr.
